# Coronary calcium in patients with and without diabetes: first manifestation of acute or chronic coronary events is characterized by different calcification patterns

**DOI:** 10.1186/1475-2840-12-161

**Published:** 2013-11-05

**Authors:** Joseph Shemesh, Alexander Tenenbaum, Enrique Z Fisman, Nira Koren-Morag, Ehud Grossman

**Affiliations:** 1 Cardiac Rehabilitation Institute, Sheba Medical Center, 52621 Tel-Hashomer, Israel; 2 Sackler Faculty of Medicine, Tel-Aviv University, 69978 Tel-Aviv, Israel; 3 Cardiovascular Diabetology Research Foundation, 58484 Holon, Israel

**Keywords:** Angina pectoris, Atherosclerosis, Coronary calcification, Coronary artery disease, Coronary computed tomography, Diabetes mellitus, Myocardial infarction

## Abstract

**Background:**

Coronary artery calcification (CAC) is closely related to coronary atherosclerosis. However, less is known about the clinical significance of extensive CAC (ECAC) in regard to types of first coronary events (acute vs. chronic). Diabetes mellitus (DM) represents a strong risk factor for CAD although its association with CAC is controversial. Aiming to elucidate these controversies we investigated the long-term outcome of coronary artery disease (CAD) in relation to degree of CAC in patients with and without DM from our annual cheek-up outpatient clinic.

**Methods:**

Coronary artery computed tomography (CT) was performed in 667 patients who were yearly evaluated during a mean follow-up period of 6.3 ±3.4 year. The following 4 CAC categories were established: calcium absence; total calcium score (TCS): 1–100 AU; TCS: 101–600 AU and ECAC: TCS above 600 AU. Acute event was defined as first acute myocardial infarction (MI) or a new unstable angina. First chronic event was defined as a positive stress test with a consequent elective percutaneous coronary intervention or coronary artery bypass grafting.

**Results:**

628 subjects (94%) were free from any cardiac events, 39 (6%) experienced first cardiac event: 18 of them suffered acute and 21 chronic events. There were 67 patients with and 600 patients without DM: 78% of patients with DM presented CAC vs. 50% of patients without DM (p < 0.001).The mean TCS was 17 times higher in the chronic than in the acute events group: 914 vs. 55 AU, p < 0.001. In 95% of the patients with chronic events more than one calcified vessel was found, compared to 67% of the patients with acute events and only 30% of those without events (p < 0.001). Incidence of CAD events (all types pooled together) rose consequently from 2% in subjects without CAC to 34% in subjects with ECAC (p < 0.001). However, among the 32 subjects with ECAC, 11 (34%) developed chronic event while none of them had acute event. In contrast, none of subjects with TCS =0 or TCS 1–100 AU presented with chronic events. Subjects with TCS 101–600 AU presented 10 (9%) chronic and 5 (4.5%) acute events (p < 0.001).

**Conclusions:**

Asymptomatic subjects with ECAC are not firstly manifested as acute coronary events but presented a high level of chronic CAD-related events during the 6.3 ±3.4 year follow-up. In contrast, first acute CAD-related events occurred mostly in subjects with mild and moderate CAC score.

## 

Coronary artery calcification (CAC) is a well established surrogate marker of the total burden of coronary atherosclerosis [1-3]. Large prospective studies consistently demonstrated that higher CAC scores are associated with increased risk for coronary artery disease (CAD) related events [4,5] and that addition of CAC score to traditional risk factors improves risk stratification [5,6]. Previously, we [7,8] and others [9] have suggested that subjects with extensive CAC (ECAC) are at increased risk of stable angina but not of acute coronary syndrome, compared to the those with the lower CAC scores.Diabetes mellitus (DM) represents a strong risk factor for CAD, albeit its association with CAC is controversial. While a number of studies have found significant relationship between high CAC and DM [10-13], the Multi-Ethnic Study of Atherosclerosis (MESA) did not confirm this association [9]. In an aim to elucidate these controversies we sought to investigate the long-term CAD outcome (stratified according to chronic or acute events) in relation to degree of CAC in patients with and without DM undergoing our annual cheek-up outpatient clinic.

## Material and methods

### Patient selection

Seven hundred and forty five consecutive subjects who consented to perform a cardiac computed tomography (CT) for coronary calcium evaluation were recruited from our annual cheek-up clinic between January 2001 and January 2002. The inclusion criteria for screening were men above 40 and women above 50 years free of cardiovascular disease. Those who had at least one year of follow-up were included in the present analysis. Seventy six subjects who never returned to the annual check-up were excluded and two others were excluded since an acute MI was found in their files before the study entry. The remaining 667 subjects comprised our study group. All underwent CT at baseline visit and were then yearly evaluated. The development of events was finally assessed on February 2011. So, the follow-up period was until the first diagnosis of event, death, last annual check –up, or 31 January 2011, providing a maximum follow-up of 10 years, mean 6.3 ±3.4 years.

### Events definition

Only first coronary events were analyzed. We defined acute event as first acute MI or hospitalization for a new unstable angina. Chronic event was defined as positive stress test on the routine annual check-up, with or without chest pain, with a consequent elective coronary catheterization resulting in percutaneous coronary intervention (PCI) or coronary artery bypass grafting (CABG).

### Follow-up

Events were recorded from the files of the annual check-up program in our institute and were verified by the available documentation. Data on mortality and cause of death were available for all participants from the registry of the Ministry of Internal Affairs. A written informed consent was obtained from all patients and the entire study protocol was approved by the Sheba Medical Center Helsinki Committee.

### Annual check-up

All subjects were clinically evaluated at baseline and then annually, and a detailed medical history and standard physical examination were performed. The following variables were recorded: age, height, weight, concomitant diseases, smoking habits, and laboratory evaluation. Estimated glomerular filtration rate (eGFR) was calculated according to the Chronic Kidney Disease Epidemiology Collaboration (CKD-EPI) equation [14].

### Coronary CT

All CT scans were performed on a dual-detector spiral CT without ECG gating and without contrast injection. Scanning protocol and CAC measuring were done according to a previously published protocol using the modified Agatston method [15]. Total CAC score was the sum of all the individual calcific lesions identified within the area of the coronary arteries. The reproducibility of calcification scoring by this method is high, with an intra class correlation coefficient of 0.99 and of 0.94 for inter-observer agreement [16]. The following 4 CAC categories were distinguished: absence of CAC; mild-moderate CAC: 1–100 AU; high CAC: 101–600 AU and very high ECAC score: above 600 AU, which corresponds to the 95^th^ percentile in our cohort.

A TCS > 0 was considered positive for the presence of any CAC. The number of coronary vessels with CAC was recorded for each subject and defined as having at least one calcific lesion in one of the 4 regions: main left artery, left anterior descending artery (LAD) and its branches, left circumflex artery (LCX) and its branches and right coronary artery (RCA) and its branches.

### Assessment of cardiovascular risk factors

Height and weight were measured with participants wearing light clothing without shoes. The body mass index (BMI) was calculated as weight (Kg) divided by the square of the height. Blood pressure (BP) was measured in the seated position after 3 minutes of rest. Hypertension was defined when BP levels were twice ≥140 mm Hg for systolic BP and/or ≥90 mm Hg for diastolic BP or a history of hypertension was reported or when the subject was on antihypertensive medications. Diabetes mellitus (DM) was defined when fasting plasma glucose was on 2 occasions greater than 126 mg/dL (7.0 mmol/L), a history of DM was reported or when the patient was on insulin or oral hypoglycemic medications. Hypercholesterolemia was defined when measured total cholesterol was > 250 mg/dl, or when the patient reported medication with cholesterol lowering agents. Smoking status was determined according to the questionnaire.

### Statistical methods

Data were analyzed with IBM SPSS software version 21.0 and were presented as frequencies and percentages for categorical variables and as mean and standard deviation for continuous variables. Significance levels were set at 0.05. The distributions of continuous variables in the study were examined using the Kolmogorov-Smirnov non-parametric test. To demonstrate the differences between the event groups (no event, chronic event and acute event), Chi-Square tests were analyzed for differences in categorical baseline characteristics and incidence of events between the groups. One-way ANOVA tests were performed for continuous characteristic including TCS values, following by analysis of covariance (ANCOVA) to adjust for age as a potential covariate.

## Results

The mean period of follow-up was 6.3 ±3.4 year. During this period 628 subjects (94%) were free from any cardiac events (Group I). Throughout follow up 39 (6%) of patients experienced a cardiac event: 18 of them suffered acute events (13 acute MI and 5 unstable angina pectoris – Group II) and in 21 of them chronic events were documented (Group III). Eight patients died without having any known prior cardiac event (non-cardiac death).

The baseline characteristics of the participants are given in Table 1. Patients with events were older than those without and those with chronic events were older than those with acute. The frequency of hypertension was twice higher among those with chronic than in those with acute event and no events. All the other clinical and laboratory parameters were not statistically different between study groups. There were 67 patients with and 600 patients without diabetes in our study. It should be pointed out that 78% of the patients with DM had CAC (TCS above zero) vs. 50% of patients without DM (p < 0.001).

**Table 1:** Baseline characteristics of the study population

**Characteristics**	**No event N = 628**	**Acute event N = 18**	**Chronic event N = 21**	**p value**
Age	55 ± 7.2	57 ± 8.2	60 ± 7.7	0.007
Male gender	526 (84)	17 (94)	21 (100)	0.073
BMI (kg/m2)	27.0 ± 3.5	27.7 ± 4.0	26.2 ± 2.7	0.395
SBP (mmHg)	126 ± 17	124 ± 15	130 ±13	0.444
DBP (mmHg)	79 ± 9	78 ± 9	79 ± 6	0.906
Hypertension	163 (26)	5 (28)	12 (57)	0.007
Diabetes	60 (10)	3 (17)	4 (19)	0.232
Current smokers	106 (22)	4 (33)	5 (31)	0.454
Chronic renal failure	14 (2)	1 (6)	1 (5)	0.511
CAD family history	162 (26)	3 (18)	7 (35)	0.486
Hyperlipidemia	284 (47)	9 (50)	12 (60)	0.475
Urea (mg/dl)	33 ± 7.7	35 ± 7.7	32 ± 5.7	0.557
Creatinine (mg/dl)	1.07 ± 0.15	1.08 ± 0.17	1.13 ± 0.11	0.179
Glucose (mg/dl)	99 ± 23	96 ± 18	101 ± 21	0.761
Calcium (mg/dl)	9.6 ± 0.45	9.6 ± 0.46	9.6 ± 0.50	0.908
Phosphorus (mg/dl)	3.02 ± 0.50	2.76 ± 0.27	3.0 ± 0.69	0.088
Triglyceride (mg/dl)	143 ± 79	156 ± 63	133 ± 58	0.671
Total cholesterol (mg/dl)				
HDL-cholesterol (mg/dl)	45 ±12	40 ± 5	45 1 ± 3	0.243
LDL-cholesterol (mg/dl)	127 ± 29	127 ± 37	127 ± 26	0.999

Data are number (%) of patients or mean ± SD.

The CAC characteristics of patients are given in Table 2. Among subjects without events half had CAC (TCS > 0) while all those with chronic and three quarter of the acute events group had CAC. Equally, the mean CAC score was 10 times higher in the chronic events group than in those without events (914 vs. 93 AU) and 17 times higher than the mean TCS of the acute group (914 vs. 55 AU). Ninety five percent of the patients with chronic events had more than one calcified vessel compared to 67% of the patients with acute events and only 30% of those without events (p < 0.001).

**Table 2:** The CAC characteristics according to outcome during follow-up

**Variable**	**No event N = 628**	**Acute event N = 18**	**Chronic event N = 21**	**p value**
TCS mean (SD)	93 ± 276	55 ± 62	914 ± 660	<0.001
TCS >0	311 (50)	13 (72)	21 (100)	<0.001
CV > 1	187 (30)	12 (67)	20 (95)	<0.001
No CV mean (SD)	1.02 ± 1.15	1.72 ± 1.23	3.05 ± 0.74	<0.001

TCS - Total calcium score (AU); CV - calcified vessel >1 - more than one calcified major coronary vessel in one of the 4 regions: main left artery, LAD, LCX and RCA including their branches; No CV – mean number of calcified major coronary vessels.

For TCS >0 and CV > 1 data are number (%) of patients.

**Figure 1:**
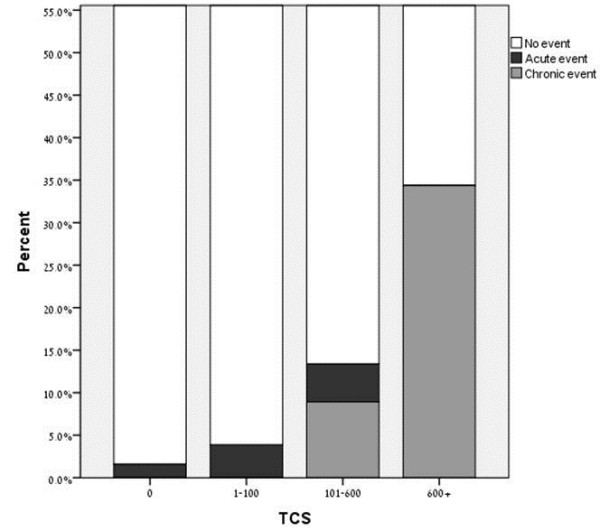
**Incidence of acute and chronic events by TCS categories.** TCS - Total calcium score (AU).

The incidence of acute and chronic events by TCS categories is given in Table 3 and Figure 1. During follow-up incidence of the CAD events (all types pooled together) rose consequently from 2% in subjects without CAC to 34% in subjects with the highest CAC category TCS > 600 AU (p < 0.001). However, different pattern in the development of the first acute and chronic coronary events in accordance with the CAC category was observed. Among the 32 subjects with TCS > 600 AU, 11 (34%) developed chronic event while none of them had acute event. In contrast, none of subjects with TCS = 0 or TCS 1–100 AU had chronic events. Subjects with TCS 101–600 AU presented 10 (9%) chronic and 5 (4.5%) acute events (p < 0.001).

**Table 3:** Incidence of acute and chronic events by TCS categories

**Type of event**	**TCS = 0**	**TCS 1–100**	**TCS 101–600**	**TCS > 600**	**p value**
	**N = 315**	**N = 208**	**N = 112**	**N = 32**	**(age-adjusted)**
All types	5 (2)	8 (4)	15 (13.5)	11 (34)	<0.001
Acute	5 (2)	8 (4)	5 (4.5)	0	
Chronic	0	0	10 (9)	11 (34)	

Data are number (%) of patients.

TCS - Total calcium score (AU).

## Discussion

The main finding of our study is that asymptomatic subjects with ECAC, both with and without DM did not developed first acute CAD-related events during up to 10 years of follow-up. Our data support the suggestion that ECAC is associated with a stable form of atherosclerosis which is mainly manifested by a chronic rather than acute course of CAD [7-9].

Previous studies have shown a dramatically increased incidence rate of CAD events (when all types of events pooled together) among those with the highest levels of CAC compared to those with CAC = zero [3-6]. However, as we demonstrate in the current analysis, a different pattern in development of acute and chronic coronary events in accordance with CAC category can be distinguished. The acute events in our study occurred mostly in subjects with mild and moderate CAC and even in absence of CAC. This might have an important clinical relevance, since minimal or mild CAC should not be categorized into a lower risk category (as suggested by the SHAPE investigators) [17]. These findings are also supported by histopathological studies, intravascular ultrasound and CT observations that consistently demonstrated that acute events rise mainly from soft non-calcified or mildly calcified plaques [18-27]. In a series of cases of sudden coronary death, more than 50% of thin-cap fibroatheromas showed a lack of calcification or only speckled calcification on postmortem radiographs of coronary arteries [18]. Several studies demonstrated the destabilizing effect of the lipid core, whereas presence of calcium was a stabilizing force and calcified lesions were more resistant to rupture (similar to fibrous plaque) [19,20].

Thus, it seems that ECAC represents a long standing chronic stage of slowly growing coronary atherosclerosis and can be regarded as a healing process leading to stabilization and decrease vulnerability of the plaques [28]. On the other hand, ECAC causes negative vessels remodeling resulting in flow restriction and leading to clinical manifestations such as chronic angina or silent ischemia upon a routine stress test [18-28].

The prevalence of CAC in our patients with DM was significantly higher than in patients without DM. The clinical value of CAC score in diabetic patients has been recently demonstrated in several studies: CAC score can further stratify diabetic patients according to the presence and extent of CAC into patients with very low or high CV risk [10-13,29].

It should be stated that our population was predominantly masculine, and this may represent a potential limitation since the epicardial adipose tissue volume shows gender disparities, being strongly associated with coronary atherosclerosis in men [30]. Therefore, despite of the strong theoretical background and biological plausibility, caution should be used in interpreting our findings because it was not a randomized controlled trial and we cannot rule out other factors that could have influenced the observed clinical outcomes.

## Conclusions

Asymptomatic subjects with ECAC did not develop first acute events, but presented a high level of chronic CAD-related events during a long term follow-up. In contrast, most of the acute CAD-related events firstly occurred in subjects with mild and moderate CAC.

## Abbreviations

AU: Agatston units; BP: Blood pressure; BMI: Body mass index; CABG: Coronary artery bypass grafting; CAD: Coronary artery disease; CAC: Coronary artery calcification; CT: Computed tomography; DM: Diabetes mellitus; ECAC: Extensive coronary artery calcification; LAD: Left anterior descending artery; LCX: Left circumflex artery; MI: Myocardial infarction; PCI: Percutaneous coronary intervention; RCA: Right coronary artery; TCS: Total calcium score.

## Competing interests

All authors declare that they have no competing interests.

## Authors’ contributions

JS and EG conceived the study, JS, AT and EZF drafted the manuscript, JS, AT, EG and NKM were involved in the study design, coordination and data acquisition, JS and NKM studied and matched the records from the computed Registry, EZF, AT, JS and EG interpreted the results, NKM performed the statistical analysis of the data presented, AT, EZF and EG critically reviewed the study for important intellectual content. All authors approved the final version of the manuscript.
